# Case Report: Development of a thyrotropinoma in a patient with thyroid hemiagenesis

**DOI:** 10.3389/fendo.2026.1902011

**Published:** 2026-08-27

**Authors:** Emil Bartosz Rozenek, Mark Gurnell, Jacek Kunicki, Maria Maksymowicz, Marta Korbonits, Wojciech Zgliczyński, Maria Stelmachowska-Banaś

**Affiliations:** 1 Department of Endocrinology, Centre of Postgraduate Medical Education, Warsaw, Poland; 2 Doctoral School of Translational Medicine, Centre of Postgraduate Medical Education, Warsaw, Poland; 3 Cambridge Endocrine Molecular Imaging Group, Metabolic Research Laboratories, Institute of Metabolic Science, University of Cambridge, and National Institute for Health Research Cambridge Biomedical Research Centre, Addenbrooke’s Hospital, Cambridge, United Kingdom; 4 Department of Neurosurgery, Maria Sklodowska-Curie National Research Institute of Oncology, Warsaw, Poland; 5 Department of Cancer Pathomorphology, Maria Sklodowska-Curie National Research Institute of Oncology, Warsaw, Poland; 6 Centre for Endocrinology, Barts and The London School of Medicine, Queen Mary University of London, Charterhouse Square, London, United Kingdom

**Keywords:** hyperthyroidism, inappropriate TSH secretion, thyroid dysgenesis, thyroid hemiagenesis, thyrotropinoma, thyrotropin-secreting pituitary adenoma

## Abstract

Thyroid hemiagenesis (THA) is a relatively rare disorder in which one thyroid lobe fails to develop. Although most individuals remain clinically euthyroid, they typically exhibit higher thyrotropin (TSH) concentrations than those with a bilobed thyroid gland, possibly reflecting reduced thyroidal reserve and a chronic compensatory response by pituitary thyrotrophs. While pituitary hyperplasia has been reported in the setting of hypothyroidism in THA, a TSH-secreting pituitary neuroendocrine tumor (thyrotropinoma) has not previously been described in this condition. A 30-year-old woman underwent thyroid function testing during the first trimester of her first pregnancy, which revealed a TSH concentration of 2.57 mIU/L. In accordance with local clinical practice, thyroid ultrasonography was performed and demonstrated absence of the left thyroid lobe, consistent with THA. Given the TSH level >2.5 mIU/L and the potentially limited thyroidal functional reserve associated with THA, subclinical hypothyroidism was diagnosed. To prevent the development of overt hypothyroidism during pregnancy and its associated maternal and fetal complications, levothyroxine therapy was initiated. Over the following years, levothyroxine was progressively uptitrated, but despite development of clinical features of thyrotoxicosis, with raised free thyroid hormone levels, serum TSH remained unsuppressed, prompting evaluation for this discordant thyroid function pattern. Pituitary MRI demonstrated an 11mm macroadenoma. Further investigations revealed an elevated alpha-glycoprotein subunit (α-GSU) level and an attenuated TSH response to TRH stimulation. A trial of a depot somatostatin receptor ligand resulted in a marked reduction in thyroid hormone levels. Together, these findings supported the diagnosis of a thyrotropinoma. Transsphenoidal surgery was performed, and histology confirmed a PIT1 lineage plurihormonal tumor with predominant TSH expression. Following surgery, the patient developed central hypothyroidism and was recommenced on levothyroxine therapy. We report the first documented case of a pituitary adenoma in a patient with THA; notably, the lesion was the rarest pituitary tumor subtype, a thyrotropinoma. This observation raises the possibility of a biological link driven by chronic thyrotroph stimulation, potentially contributing to adenoma formation. A similar feedback-driven mechanism has been proposed to explain cases of coexistence of resistance to thyroid hormone β (RTHβ) and thyrotropinoma. Therefore, in patients with THA receiving levothyroxine therapy, the development of hyperthyroxinaemia with a non-suppressed TSH should prompt consideration of a thyrotropinoma.

## Introduction

Thyroid hemiagenesis (THA) is a relatively rare congenital disorder characterized by the absence of one thyroid lobe, with or without the isthmus. The reported prevalence is approximately 0.02–0.05%, but the true frequency remains uncertain, as many cases occur in asymptomatic, euthyroid subjects and may therefore go unrecognized (1). Most reported cases are sporadic, although familial clustering has also been described. The diagnosis can be reliably established by combining ultrasonography with thyroid scintigraphy (2). Most patients are clinically euthyroid, with normal circulating free thyroxine (fT4) levels. However, individuals with THA frequently have higher serum thyroid-stimulating hormone (TSH, thyrotropin) and free triiodothyronine (fT3) levels compared with subjects with a normally developed thyroid gland. This hormonal pattern likely indicates a limited functional reserve of the single-lobed thyroid, and a compensatory response to maintain euthyroidism (2–4). Chronic compensatory stimulation of pituitary thyrotrophs could theoretically predispose to thyrotroph hyperplasia, which has indeed been described in the setting of THA associated with hypothyroidism. To our knowledge, however, coexistence of THA with a pituitary adenoma has not been reported.

TSH-secreting pituitary neuroendocrine tumors (thyrotropinomas) (5), are a rare but important cause of central hyperthyroidism (i.e., high free thyroid hormone levels with non-suppressed TSH), and must be distinguished from non-tumoral inappropriate secretion of TSH (IST) caused by the genetic disorder resistance to thyroid hormone beta (RTHβ) (6). In thyrotropinomas, autonomous TSH secretion is unresponsive to the normal T3-mediated negative feedback, resulting in hyperthyroxinaemia due to continued inappropriate TSH secretion. Symptoms of hyperthyroidism, which are present in approximately 75% of cases, are often milder than those observed in primary hyperthyroidism (7, 8). However, because many thyrotropinomas present as macroadenomas (often with local invasion), 30-40% of patients manifest compressive symptoms including visual field defects (9). Their prevalence is estimated at only 1–3 cases per million in the general population, and they account for approximately 0.5% to 2% of all pituitary adenomas (7). The molecular mechanisms driving thyrotrope adenomatous transformation remain largely unknown (9).

Here we report the first case of a thyrotropinoma arising in a patient with THA. As both conditions are rare, their coexistence may signify a link between prolonged thyrotroph stimulation and development of an autonomous tumor.

## Case description

A 36-year-old woman was referred to the Endocrinology Department because of progressive symptoms of hyperthyroidism accompanied by discordant thyroid function tests (TFTs): elevated free thyroid hormone levels (hyperthyroxinaemia) with a non-suppressed TSH.

**Figure 1:**
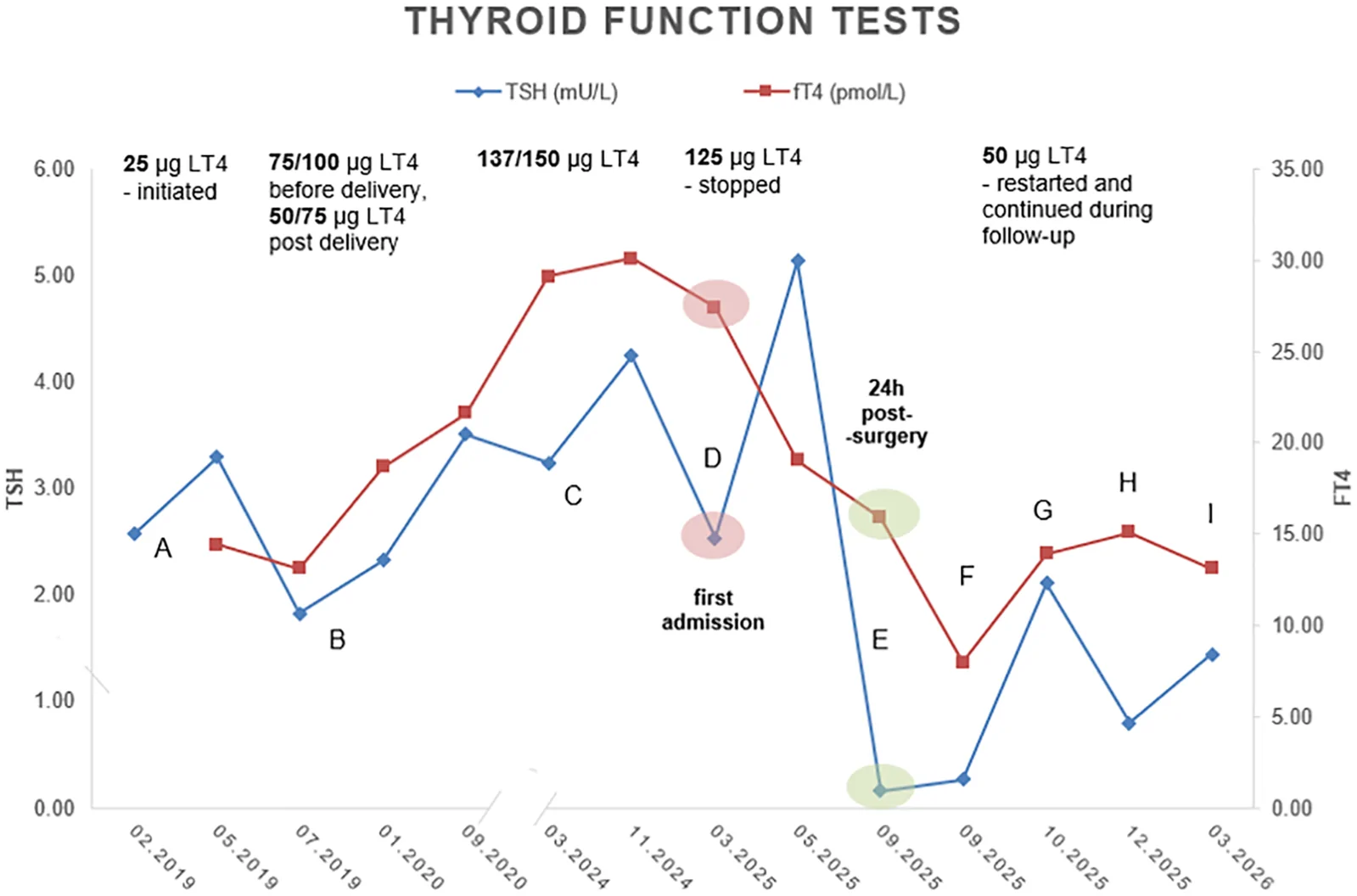
Serial thyroid function tests. **(A)** During the first trimester of first pregnancy, hypothyroidism was diagnosed, and ultrasonography revealed agenesis of the left thyroid lobe. Levothyroxine therapy was initiated at 25 µg/day and subsequently titrated to 75 and 100 µg on alternate days. **(B)** Vaginal delivery (10.2019). After pregnancy, the levothyroxine dose was reduced to 50 and 75 µg on alternate days. The X-axis break denotes the period between 09.2020 and 03.2024 (not shown), during which progressively higher doses of levothyroxine were administered due to a gradual increase in TSH levels. The treatment was initially well tolerated. **(C)** The patient was receiving levothyroxine at a dose exceeding standard weight-based replacement requirements (137 and 150 µg on alternate days at a body weight of 65 kg), which resulted in elevated serum thyroid hormone levels and a gradual deterioration in well-being accompanied by symptoms of hyperthyroidism (03.2024). **(D)** Hospitalization; levothyroxine was discontinued (the patient had been receiving 125 µg daily). **(E)** 24 hours after transsphenoidal surgery (TSS). **(F)** 7 days after TSS – levothyroxine was reintroduced at a dose of 50 µg daily. **(G)** 3 weeks after TSS. **(H)** 3 months after TSS – levothyroxine 50 µg daily. **(I)** 6 months after TSS – levothyroxine 50 µg daily. LT4, levothyroxine.

Five years earlier, during the first trimester of her first pregnancy, TFTs were performed as part of routine early-pregnancy screening in Poland. Laboratory evaluation showed a TSH level of 2.57 mU/L [first-trimester Poland-specific reference range: 0.009-3.18 mU/L (10)]. In accordance with local clinical practice standards, thyroid ultrasonography was subsequently performed. The examination visualized only the right lobe and isthmus, raising suspicion of THA. At that time, neither biochemical nor ultrasound features of autoimmune thyroiditis were present. Because pregnancy increases thyroid hormone requirements and THA may limit thyroidal functional reserve (3), low-dose levothyroxine therapy was initiated to mitigate the risk of subsequent overt hypothyroidism and its associated complications (11). A reduction in TSH was achieved only after gradual levothyroxine dose escalation, with free thyroid hormone concentrations remaining stable and within the reference range throughout the remainder of the pregnancy (**Figures 1A, B**).

**Figure 2:**
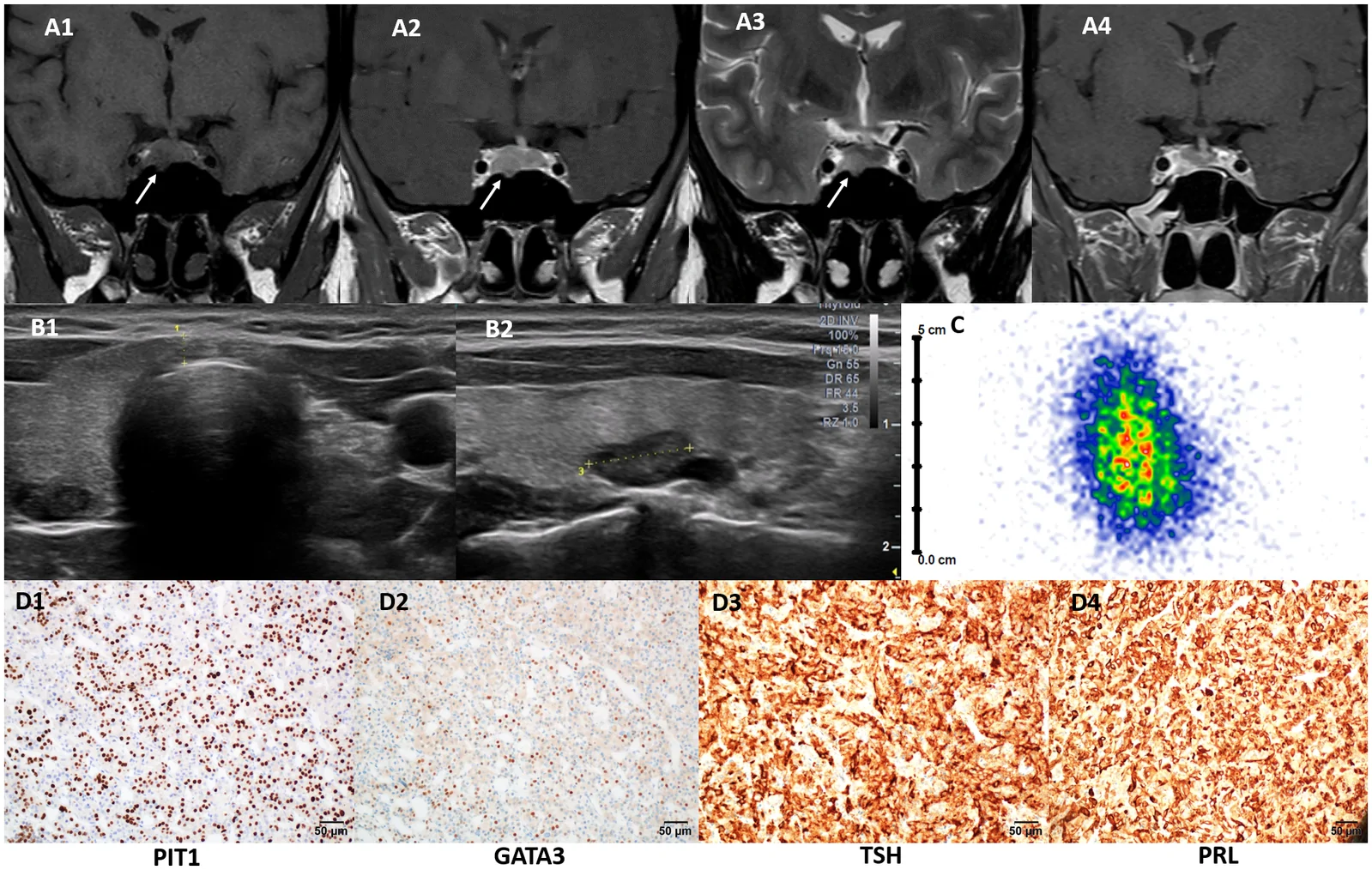
Imaging findings demonstrating pituitary macroadenoma (MRI) and thyroid hemiagenesis (ultrasonography; iodine scintigraphy) and immunohistochemistry of pituitary tumor. Pituitary MRI **(A1)**, coronal pre-contrast T1-weighted image; **(A2)**, coronal post-contrast T1-weighted image; **(A3)**, coronal T2-weighted image; **(A4)**, three months after pituitary surgery coronal post-contrast T1-weighted image. Thyroid ultrasonography **(B1)**, transverse view; **(B2)**, longitudinal view). Thyroid iodine scintigraphy **(C)**. Images **(A1**, **A2**, **A3)** demonstrate a non-invasive pituitary macroadenoma measuring 10 × 7 × 11 mm (white arrow), with a 3-mm leftward deviation of the pituitary stalk. Image **(A4)** demonstrates a gross total resection of the pituitary macroadenoma. Immunohistochemistry of PIT1 lineage plurihormonal PitNET with predominant TSH expression and focal PRL positivity **(D1)**, nuclear reactivity for PIT1; **(D2)**, nuclear reactivity for GATA3; **(D3)**, positive expression of TSH; **(D4)**, positive expression of PRL. In the thyroid evaluations, absence of the left thyroid lobe with a normally developed right lobe and isthmus was observed, consistent with left-sided thyroid hemiagenesis **(B1, C)**. On ^131^I scintigraphy, the characteristic “hockey stick sign” is visible **(C)**, representing the most common anatomical pattern of thyroid hemiagenesis (1). Image **(B2)** demonstrates solid 12×8×7 mm thyroid nodule with a polylobulated contour (EU-TIRADS-PL 5), which proved benign on fine-needle aspiration cytology (Bethesda II).

Following delivery, the levothyroxine dose was reduced to 50 and 75 μg on alternate days, with satisfactory biochemical and clinical responses. However, over the next few years progressively higher doses of levothyroxine were prescribed in response to a gradual rise in TSH levels. While the dose escalation was initially well tolerated, levothyroxine administration exceeding standard weight-predicted requirements resulted in elevated serum thyroid hormone concentrations and hyperthyroid symptoms (including heat intolerance, paroxysmal palpitations, emotional lability, insomnia, and hair loss) (**Figure 1C**). In the absence of an alternative explanation for these discordant TFTs, pituitary magnetic resonance imaging (MRI) with gadolinium enhancement was performed. This demonstrated a pituitary macroadenoma measuring 10 × 7 × 11 mm (**Figures 2A1, A2, A3**). The patient reported frequent headaches, but denied visual disturbances, or other local symptoms related to the pituitary macroadenoma. Testing for thyrotropin receptor antibodies (TRAb) was negative, ruling out concomitant Graves’ disease. The patient was referred to our Endocrinology Department for further evaluation and management (**Figure 1D**).

## Diagnostic assessment

The patient’s laboratory results at admission are summarized in **Table 1**. Analytical interference was excluded, and no transient physiological or clinical conditions that could account for the abnormal TFTs were identified. Although progressive escalation of levothyroxine resulted in a partial decrease in serum TSH, this occurred at the expense of supraphysiological thyroid hormone concentrations and clinical symptoms of hyperthyroidism.

**Table 1:** Laboratory results at first hospital admission and 3 months after transsphenoidal surgery.

Lab test	Before surgery	3 months after surgery	Unit	Reference range
**TSH**	**2.531**	0.802	**mU/L**	**0.35–4.00**
**fT4**	**27.41**	15.07	**pmol/L**	**10.29–22.70**
**fT3**	**8.23**	4.67	**pmol/L**	**3.53–6.45**
SHBG	113.8 137.4	78	nmol/L	48.2–137.2
IGF-1	30.59	28.50	nmol/L	10.48–36.29
GH	0.74	0.36	µg/L	0.10–6.88
FSH	7.8	6.6	U/L	2.8–10.2
LH	3.2	4.3	U/L	1.1–9.2
Estradiol	263.95	294.33	pmol/L	0–411.15
PRL	555.44 521.28	314.89	mU/L	40.28–530.0
ACTH	2.33	1.25	pmol/L	1.10–10.13
Cortisol 8 a.m.	300.69	460.69	nmol/L	137.95–689.75
anti-Tg	<15		kU/L	0–60
anti-TPO	51.8		kU/L	0–60
Insulin	36.81	29.17	pmol/L	13.89–152.79
Glucose	5.89	4.89	mmol/L	3.89–5.50
HbA1c	32		mmol/mol	<38.8
**α-GSU**	**1.11** **1.24**	0.33	µg/L	**0.0–0.9**
β-hCG	<0.2	<0.2	U/L	<5
CRP	<0.6		mg/L	0–5
Creatinine	58.34	67.19	µmol/L	44.20–79.56
ALT	0.306		µkat/L	0.167–0.585

TSH, thyroid-stimulating hormone; fT4, free thyroxine; fT3, free triiodothyronine; SHBG, sex hormone-binding globulin; IGF-1, insulin-like growth factor 1; GH, growth hormone; FSH, follicle-stimulating hormone; LH, luteinizing hormone; PRL, prolactin; ACTH, adrenocorticotropic hormone; anti-Tg, anti-thyroglobulin antibodies; anti-TPO, anti-thyroid peroxidase antibodies; HbA1c, glycated hemoglobin; α-GSU, alpha-glycoprotein subunit; β-hCG, beta-human chorionic gonadotropin; CRP, C-reactive protein; ALT, alanine aminotransferase. Bold values denote key measurements discussed in the text.

Based on these findings, IST was confirmed, and further investigations were directed towards differentiating between a thyrotropinoma and RTHβ. The diagnostic evaluation is summarized in **Table 2**. Collectively, the biochemical findings, the presence of a pituitary macroadenoma, and the absence of IST in the patient’s parents supported the diagnosis of thyrotropinoma. Given the euthyroid state after levothyroxine withdrawal, the patient did not require preoperative preparation with somatostatin receptor ligand (SRL). However, it was initiated to further support the preliminary diagnosis. Follow-up assessment was performed 2 weeks after administration of lanreotide autogel 120 mg subcutaneously. TFTs demonstrated TSH deficiency resulting in biochemical hypothyroidism, consistent with the patient’s reported symptoms. In the context of the overall clinical, hormonal and radiological findings, the patient was referred for elective transsphenoidal surgery (TSS).

**Table 2:** Diagnostic differentiation between thyrotropinoma and RTHβ in the setting of inappropriate TSH secretion.

Assessment	Result	Interpretation
α-GSU	α-GSU (µg/L): 1.24	elevated (> 0.9 µg/L)
TSH response to TRH stimulation test ^a^	TSH (mU/L): 3.716 baseline; 9.581 peak	blunted (<3 fold of baseline) ^b^
short trial of long-acting SRL ^c^	TSH (mU/L): 0.162 ^d^ fT3 (pmol/L) 1.83 fT4 (pmol/L) 4.76	positive (marked reduction of fT3 and fT4) ^e^
SHBG	SHBG (nmol/L) 1) 113.8 (normal-high) 2) 137.4 (elevated)	variable (normal to elevated)

Reference ranges are listed in **Table 1**.

^a^ TRH (thyrotropin-releasing hormone; protirelin, TRH Ferring) 200 µg was administered intravenously. TSH was measured at baseline (0 min, before injection) and at 20, 60, 90 and 120 minutes after injection.

^b^ Blunted = TSH increment < 3-fold of baseline; suggestive of a thyrotropinoma. A 2.58-fold TSH increase was observed, fulfilling the criterion for a blunted TRH stimulation response.

^c^ SRL (somatostatin receptor ligand).

^d^ Values presented in the table refer to measurements obtained 2 weeks after lanreotide autogel 120 mg SC administration.

^e^ The last laboratory assessment prior to lanreotide administration (>1 month after levothyroxine withdrawal) showed: TSH (mU/L) 5.14; fT3 (pmol/L) 6.05; fT4 (pmol/L) 19.1, therefore, the marked reduction in fT3 and fT4 during the test was very suggestive of a thyrotropinoma.

In parallel with the endocrine evaluation, thyroid ultrasonography revealed a 12×8×7 mm focal lesion with a high risk of malignancy (EU-TIRADS-PL 5) in the right thyroid lobe (**Figures 2B1, B2**). The lesion had previously been detected during pregnancy but did not exhibit suspicious sonographic features at that time, and fine-needle aspiration cytology (FNAC) was benign. Given literature reports suggesting an increased risk of thyroid cancer not only in thyroid hemiagenesis (THA) (2, 3) but also in thyrotropinoma (9), FNAC was repeated.

Cytological evaluation again confirmed a benign lesion (Bethesda II). The patient was advised to undergo regular ultrasound surveillance of the thyroid nodule. As part of the diagnostic workup, ^131^I thyroid scintigraphy was performed after withdrawal of levothyroxine, when the patient was biochemically euthyroid. No radiotracer uptake was observed in the expected anatomical location of the left thyroid lobe, consistent with the ultrasound findings and confirming left-lobe THA. Additionally, no focal area of increased or decreased tracer uptake corresponding to the ultrasound-described lesion was identified (**Figures 2B1, B2, C**).

## Treatment

Levothyroxine therapy was initiated to correct hypothyroidism induced by the SRL trial. Thyroid hormone replacement was later withdrawn, and the patient remained euthyroid off all treatment at the time of pituitary surgery. The patient underwent transsphenoidal resection of a pituitary tumor at Bielanski Hospital in Warsaw (Poland).

## Outcome and follow up

TSS proceeded uneventfully and 24 hours after the surgery, serum TSH had fallen to 0.155 mU/L (**Figure 1E**). On day 5 postoperatively, the patient manifested clinical and biochemical TSH deficiency (fT3 1.89 pmol/L; fT4 7.98 pmol/L with an inappropriately low TSH of 0.266 mU/L) (**Figure 1F**). These postoperative results were consistent with effective surgical treatment. One week after surgery, thyroid hormone levels remained below the reference range, while TSH remained low at 0.611 mU/L. The patient reported concentration difficulties, dry skin, and hair loss. Levothyroxine therapy was initiated at a dose of 50 μg/day. By four weeks postoperatively, TSH had increased to 2.11 mU/L and thyroid hormone levels had normalized, with the latter likely reflecting the combined effect of levothyroxine replacement therapy and progressive recovery of normal thyrotroph function (**Figure 1G**). Histopathology confirmed a PIT1 lineage plurihormonal PitNET, GATA3 positive with predominant TSH expression and focal PRL positivity (**Figures 2D1–D4**), consistent with a clinically functioning thyrotropinoma. At 3 months postoperatively, the patient underwent a comprehensive reassessment of anterior pituitary function, which confirmed preserved anterior pituitary hormonal function (**Table 1**). Follow-up pituitary MRI demonstrated gross total resection of the pituitary adenoma (**Figure 2A4**). Clinically, the patient reported a marked improvement in her overall condition, with substantial resolution of the presenting symptoms. In particular, irritability, anxiety, and sleep disturbances resolved completely. Headaches became considerably less frequent and less severe, while episodes of hot flushes, excessive sweating, and palpitations also resolved. At both 3- and 6-month follow-up visits, TFTs remained within the reference range while the patient was receiving levothyroxine at a dose of 50 μg/day (**Figures 1H, I**).

## Discussion

Once genuine elevated free thyroid hormone levels with non-suppressed TSH are confirmed, the differential diagnosis lies between a thyrotropinoma and RTHβ. Distinguishing between these two conditions often requires a multimodal approach (12). In general, the coexistence of a pituitary macroadenoma and elevated serum alpha-glycoprotein subunit (α-GSU) is strongly suggestive of a thyrotropinoma (9), and in our patient was also supported by the finding of a blunted TSH response to thyrotropin-releasing hormone (TRH) stimulation (insufficient fold-increase), mildly elevated sex hormone-binding globulin (SHBG) concentration and the absence of a relevant family history. RTHβ could potentially occur *de novo*, with the pituitary lesion being incidental (6, 13). These findings can be reconciled as follows.

In general, a greater than 5-fold increase in TSH following TRH stimulation is considered supportive of RTHβ rather than thyrotropinoma, although some authors recognize a diagnostic “gray zone,” with a 3- to 5-fold TSH response being compatible with either diagnosis (6). Circulating SHBG concentrations are typically normal in RTHβ, reflecting hepatic resistance to thyroid hormone action, whereas they are generally elevated in patients with thyrotropinoma due to hepatic hyperthyroidism (6). It is important to note that not all thyrotropinomas present with a blunted TSH response to TRH stimulation or with elevated SHBG concentrations (13).

Once the working diagnosis of thyrotropinoma was established, levothyroxine therapy was immediately discontinued (**Figure 1D**). This led to free thyroid hormone concentrations returning to normal (though at the upper limit of the reference range). Furthermore, the rise in TSH after levothyroxine withdrawal revealed that the suspected adenoma retained partial feedback sensitivity, as levothyroxine had been suppressing its secretory activity to some extent. To increase diagnostic certainty, a trial with a long-acting SRL was performed, as a significant reduction in thyroid hormone levels during such a trial is not expected in RTHβ (13, 14). Thyroid function was reassessed after 2 weeks because the patient reported worsening clinical symptoms, and delaying biochemical evaluation until the full pharmacodynamic effect of lanreotide autogel had been achieved might have unnecessarily prolonged clinically significant hypothyroidism. The profound suppression of TSH together with a marked reduction in fT4 and fT3 to below the reference range strongly supported the diagnosis of thyrotropinoma.

After removal of the autonomous tumor, normally functioning thyrotrophs require time to resume their physiological activity. Thus, postoperative TSH levels are considered a marker of residual tumor activity, and this parameter is often used to assess the effectiveness of the surgical intervention (15). In a recent Korean study, the immediate postoperative TSH level (measured 12 hours after surgery) was identified as the strongest predictor of surgical cure, with an optimal cutoff of 0.62 mU/L (16). In our case, even 24 hours after surgery, the TSH concentration was even lower than the proposed cutoff. This finding can be regarded as an early marker of effective surgery and biochemical remission. Subsequent histopathological examination confirmed the diagnosis of TSH-secreting pituitary adenoma.

Mixed adenomas with concomitant hypersecretion of other anterior pituitary hormones are identified in approximately 25% of thyrotropinomas. The most frequent associations involve excess GH and/or prolactin, leading clinically to acromegaly or an amenorrhea-galactorrhea syndrome in females. This co-secretion pattern is likely related to the shared developmental lineage of thyrotrophs, somatotrophs, and lactotrophs, which differentiate via Prop-1 and PIT1-dependent pathways. Importantly, immunohistochemical positivity for additional pituitary hormones does not necessarily correspond to clinically relevant hypersecretion *in vivo* (13), a pattern also observed in the present case despite PRL positivity on immunochemistry.

THA represents a unique *in vivo* model of chronic compensatory TSH stimulation. Although serum TSH values are often within the reference range, they remain consistently higher than in individuals with bilobed thyroid glands. It has therefore been suggested that subjects with THA ought to be regarded as having long-standing subclinical hypothyroidism and as being at a higher-than-population risk of progressing to overt thyroid failure (2, 3). Patients with THA show an exaggerated TSH response to TRH stimulation, which supports the presence of underlying subclinical impairment of thyroid function (17). Hypothyroidism has been reported to occur substantially more often in individuals with THA than in euthyroid controls, whereas hyperthyroidism was observed at a comparable frequency. Nonautoimmune subclinical hypothyroidism in THA has also been reported (2, 3).

Chronic stimulation of thyrotrophs in the setting of hypothyroidism may result in secondary pituitary hyperplasia (18, 19). In bilobed thyroid glands, this phenomenon was described primarily in the context of hypothyroidism typically occurring at markedly elevated TSH concentrations. However, case reports exist in which TSH levels only slightly exceeded the upper reference limit, for example by approximately 1.5-fold. This suggests that, apart from the absolute TSH concentration, other factors may also play a role, such as individual pituitary sensitivity to TRH and the duration of stimulation (20). Over time, persistent thyrotroph stimulation may lead to nodular hyperplasia and, in rare cases, the formation of a TSH-secreting pituitary tumor, analogous to the progression observed in tertiary hyperparathyroidism. This possibility should be considered when the pituitary lesion fails to regress on thyroid hormone replacement (19, 21).

In 1985, Scheithauer et al. examined pituitary glands at autopsy in 64 patients with long-standing primary hypothyroidism. Diffuse and nodular thyrotroph hyperplasia was observed in 69% and 25% of glands, respectively, and pituitary adenoma in 18.7%. Five of the twelve adenomas (42%) contained TSH-immunoreactive cells (22). This represents a striking overrepresentation of thyrotroph adenomas relative to their prevalence in the general population (7, 23).

Most published reports of thyrotropinomas occurring in the context of primary hypothyroidism are individual case reports. Establishing whether the tumor developed as a consequence of chronic thyrotroph stimulation is difficult to demonstrate. In many patients, hypothyroidism was diagnosed promptly, and the presence of a pituitary adenoma was effectively covered up by the TFTs results. Once levothyroxine therapy failed to adequately suppress TSH, the possibility of a thyrotropinoma was considered (24, 25). A similar pattern was observed in our case (**Figure 1**). Considering that, as previously mentioned, most subjects with THA may represent a state of long-standing subclinical hypothyroidism, it is conceivable that prolonged thyrotroph stimulation could contribute to thyrotroph proliferation in some individuals. However, whether THA truly predisposes to thyrotropinoma development remains unknown. Chronic thyrotroph stimulation represents one possible mechanistic hypothesis, particularly given the congenital nature of THA.

Both THA and RTHβ may result in insufficient thyroid hormone signaling at the pituitary level, but through distinct mechanisms. In RTHβ, impaired thyrotroph sensitivity to circulating thyroid hormones leads to attenuated negative feedback, resulting in inappropriately normal or elevated TSH despite increased thyroid hormone levels (6). In THA, diminished thyroidal reserve, with reduced hormone synthesis, can result in chronically elevated TSH, signifying subclinical or overt hypothyroidism (2). Pituitary hyperplasia has been documented in both RTHβ and THA in the setting of insufficient thyroid hormone signaling to suppress TSH (18, 26, 27). Evidence from clinical reports and animal models suggests that RTHβ may predispose to the development of thyrotropinomas (28–32). Although chronic sustained thyrotroph activation could theoretically contribute to pituitary hyperplasia and, ultimately, autonomous thyrotroph proliferation, this case represents, to our knowledge, the first reported coexistence of THA and a thyrotropinoma. Notably, no pituitary adenoma of any type has previously been described in patients with THA, making this association particularly unusual. However, this observation should be regarded as hypothesis-generating, and whether the coexistence reflects a true biological association remains to be determined.

This case underscores the importance of clinical vigilance in patients with THA receiving levothyroxine therapy. Discordant TFTs suggestive of inappropriate TSH secretion should prompt consideration of early pituitary MRI to avoid neurological complications from tumor enlargement.

## Limitations

This case report has several limitations. First, as a single-case observation, it remains uncertain whether the coexistence of THA and thyrotropinoma reflects a causal relationship or merely a coincidental finding. Additional similar case reports, together with epidemiological studies comparing the prevalence of pituitary tumors in patients with THA and in individuals with a normally developed thyroid gland, are needed before these findings can be generalized. Secondly, although the patient remains in remission, continued long-term follow-up is warranted to monitor for potential tumor recurrence. Finally, although the multimodal diagnostic work-up in the present case strongly supported the diagnosis of thyrotropinoma, genetic testing for *THRB* mutations should be considered in patients with thyrotoxicosis and discordant TFTs whenever diagnostic uncertainty regarding the exclusion of RTHβ persists.

## Data Availability

The raw data supporting the conclusions of this article will be made available by the authors, without undue reservation.
